# Introduction to the symposium. Two decades of ART: success through research

**DOI:** 10.1590/S1678-77572009000700013

**Published:** 2009

**Authors:** Maria Fidela de Lima NAVARRO

**Affiliations:** DDS, PhD, Professor, Department of Dental Materials, Endodontics and Operative Dentistry, Bauru School of Dentistry, University of São Paulo, Bauru, SP, Brazil.

It gives me great pleasure, both as presidentelect of the IADR and personally, to introduce this landmark symposium celebrating two decades of Atraumatic Restorative Treatment (ART) - success through research.

While success can be defined in many ways, one definition concerns “the achievement of something desired, planned, or attempted”. In many ways this definition applies to the genesis and the evolution of the ART approach. The approach started with the desire of its originator to develop quality and appropriate oral health care which could reach those who rarely have access to care. The promising results from the early field trials then led to the planned dissemination of the ART approach in partnership with the World Health Organization, the FDI World Dental Federation and through symposia organized in collaboration with the IADR. The last and perhaps most difficult hurdle has been the attempt to awaken interest amongst the dental profession to a new way of delivering care in terms of ART and other minimal intervention approaches based on a soundly researched evidence base. Here, as we will learn in this symposium, these latter attempts are eventually succeeding.

It is important, however, to underline that this symposium not only marks the success of the ART approach itself but also marks the importance of research in achieving this success. It bears witness to how research at all levels can contribute individually to a greater whole. For example, if there had not been research which permitted us a better understanding of the caries process, research that led to improved and reliable dental materials and research that allowed the outcomes of new treatment approaches to be effectively evaluated, the ART approach would not be at the stage it is today and we would not be celebrating two decades of ART's success through research.

It is only fitting that this symposium should start with the originator of the ART approach, namely Dr. Jo Frencken (The Netherlands), describing the evolution of Atraumatic Restorative Treatment from its roots as an answer to a problem of delivery care in rural Africa where attempts to manage dental care using “traditional” approaches had failed. He will then go on to describe the establishment of field studies in Thailand and other countries to evaluate ART's potential and reliability. He will then highlight some of the many achievements over the past two decades of ART.

Passing from the macro perspective of ART to a micro perspective, Dr. Gustavo Molina (Argentina) will show the importance of basic research relating to ART covering such aspects as the importance of sealing caries lesions, fluoride release and caries remineralisation.

Moving to the patient level Dr. Soraya Leal (Brazil) will examine whether an approach which purports to be atraumatic can have an effect on patient acceptability of dental care and particularly in relation to dental anxiety and discomfort.

The oral health profession can only advance if existing and future oral health care providers are made aware of new developments and approaches and receive appropriate training so that research can be applied to day-to-day oral health practice. With respect to ART, this implies more than just technology transfer but involves the transfer of a sound understanding of the logic and research base why new approaches are necessary in oral health care. Here, as an educator and a researcher I will detail results of a study investigating the scenario of teaching ART in my native country Brasil, where two important health programs are starting to be implemented into the whole country, and the outcomes of this investigation can be helpful for the government authority in charge of these programs.

We then learn from Dr. Oswaldo Ruiz (Ecuador) how the ART approach is being incorporated into health care systems in Latin America. This is then followed by a particularly interesting and impressive country example where Dr. Heriberto Vera Hermosillo (Mexico) will detail how ART has been part of Mexico's oral health strategy for almost ten years. This has involved training of dentists, evaluation at each step of its implementation stage, and research on its effectiveness to help determine how the strategy can be improved. This is a perfect example of how research is important at every step of the oral health planning cycle and is an excellent model for other countries to follow.

Even as we celebrate two decades of ART research, the research community must not rest on its laurels but must build on the success it has achieved thus far. For this reason the symposium appropriately ends with a presentation by Dr. Christopher Holmgren (France) taking a prospective view of further research avenues relating primarily to Atraumatic Restorative Treatment but which also have applications in many other areas of caries management and oral health care.

For oral health care to improve and to become accessible to the many who do not have access or adequate access to oral health, targeted research in this important area is essential. This implies adequate funding and competent and willing research personnel. It is hoped that the publication of these symposium proceedings will stimulate all those in the research arena to take notice of the real need of research to improve oral health globally.

